# High-Frame-Rate Low-Noise Global Shutter CMOS Image Sensor for High-Speed Machine Vision †

**DOI:** 10.3390/s26041117

**Published:** 2026-02-09

**Authors:** Abhinav Agarwal, Jatin Hansrani, Kazuhisa Suzuki, Karthik Venkatesan, Wilson Law, Varun Shah, Kai Ling Ong, Danny Marine, Oleksandr Rytov, Tim Lu, Neil Kumar, Edward Enriquez, Liviu Oniciuc, Sam Bagwell, Loc Truong, Anders Andersson, Radu Corlan

**Affiliations:** 1 Forza Silicon (AMETEK Inc.), Pasadena, CA 91107, USA; 2 Forza Silicon (AMETEK Inc.), Shiba NBF Tower (1F, 3F) 1-1-30, Shiba Daimon, Minato-ku, Tokyo 105-0012, Japan; 3 Independent Researcher, Pasadena, CA 91107, USA; 4 Vision Research (AMETEK Inc.), 1 Eugen Botez St., 020232 Bucharest, Romania

**Keywords:** CMOS image sensor (CIS), global shutter pixel, high-speed machine vision, ultra-high frame rate imaging, low-noise sensor design, backside-illuminated (BSI) sensor, voltage-domain global shutter (VDGS), correlated double sampling (CDS), serializer-deserializer (SerDes) interface, industrial and scientific imaging applications

## Abstract

In this paper we present a low-noise, high-frame-rate global shutter CMOS image sensor with UHD resolution (3840 × 2160), targeting high-speed machine vision applications. The sensor (ForzaFAST581) supports video capture at up to 1141 FPS at 12 bits and 1694 FPS at 8 bits at full resolution, consuming a total power of 5.5 W. Fabricated in a 65 nm, four-metal BSI process, the imager features a 5 µm voltage-domain global shutter pixel with dual-gain capability for improved dynamic range and a read noise of 3.04 e^−^ in global shutter and 2.15 e^−^ in rolling shutter mode for high-gain at maximum frame rate operation. For compact camera integration and low power consumption, the sensor is designed to stream video through 16 CML data ports, each operating at 7.44 Gbps, achieving a total aggregate throughput of 119 Gbps. Additionally, the sensor supports selectable output bit depths—8-bit, 10-bit, and 12-bit—allowing frame rate optimization based on application-specific requirements.

## 1. Introduction

High-speed machine vision systems are increasingly deployed in applications such as industrial inspection, automotive safety testing, advanced microscopy, and slow-motion sports analysis, where capturing fast-moving subjects with high temporal resolution and minimal distortion is essential. A key enabler of these systems is the image sensor. In particular, a high-frame-rate, low-noise, global shutter CMOS image sensor is critical for accurately capturing transient events without motion artifacts or rolling shutter distortions.

While traditional rolling shutter CMOS image sensors offer advantages such as lower temporal noise and simpler pixel architecture, they suffer from temporal skew and motion blur—limitations that reduce their effectiveness in scenes with rapid motion. In contrast, global shutter (GS) pixel architectures enable true snapshot imaging by simultaneously exposing all pixels, thus eliminating temporal distortion. However, these architectures [1,2,3,4,5,6,7] often introduce trade-offs, including increased dark temporal noise, reduced fill factor, and diminished dynamic range, primarily due to the added complexity and in-pixel storage elements required for global transfer operation.

Recent advancements in pixel design, low-noise analog front ends, and high-speed ADCs have significantly closed the performance gap between global and rolling shutter image sensors. These innovations now allow GS CMOS image sensors to retain their temporal advantages while achieving competitive image quality, thereby expanding their applicability in high-performance machine vision systems where both speed and image quality are critical.

**Figure 1:**
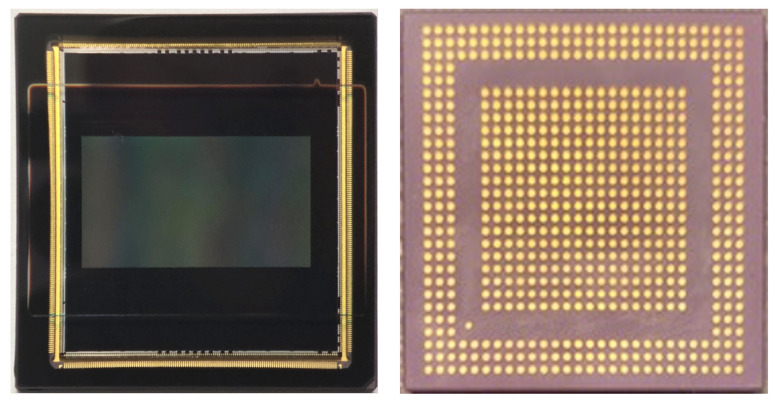
Image sensor (ForzaFAST581) die photo with 268-Pin LGA Package.

In this work, we present a high-frame-rate, low-noise global shutter CMOS image sensor optimized for high-speed machine vision applications (Figure 1). The design focuses on maximizing output frame rate while maintaining image quality through low noise, minimized horizontal smearing, and support for short integration times (<2 µs) [8].

We have chosen a voltage-domain global shutter pixel architecture over a charge domain global shutter pixel architecture in this design primarily due to the requirement of better Parasitic Light Sensitivity (PLS) [9]. The availability of the high density trench capacitor in the chosen process [10] helps to significantly minimize the pixel kTC noise, achieving noise performance more close to a charge domain global shutter pixel.

To further enhance versatility, the sensor includes column and row windowing as well as port concentration modes, which enable data throughput through a reduced number of output ports. Multiple spatial subsampling modes, such as Bayer skipping and Bayer sub-sampling, are supported to increase frame rate at lower resolutions. The readout architecture is configurable for both correlated double sampling (CDS) and non-CDS modes, allowing applications to balance noise and speed, especially during windowed readout. The sensor also supports selectable output bit depths—8-bit, 10-bit, and 12-bit—enabling additional optimization of frame rate based on application-specific requirements.

The remainder of the paper is organized as follows. Section 2 and Section 3 describe the sensor readout architecture and the row-logic implementation, respectively. Section 4 presents the high-speed SerDes architecture used for off-chip data transmission. Section 5 concludes the paper with a summary of the silicon measurement results.

## 2. Readout Architecture

**Figure 2:**
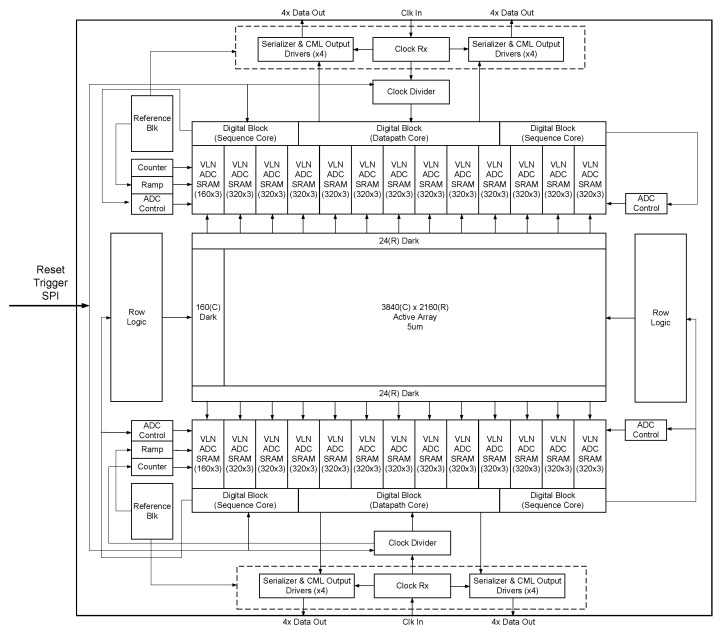
Image sensor block diagram.

The sensor block diagram (Figure 2) illustrates a top–bottom readout architecture in which six rows are read out concurrently. The pixel array readout is divided across two halves, with each side further partitioned into 12 superblocks, where each superblock reads 320 columns by 3 rows. The sensor employs a 12T voltage-domain global shutter pixel architecture with dual-gain capability. Within each pixel, two high-density deep trench capacitors (a cross-section of these deep trench capacitors is shown in [10]) store the reset and signal levels required for CDS readout. The capacitor sizes are carefully optimized to minimize kTC noise—a dominant component of overall pixel noise. A shared source follower configuration is used for pixel readout to suppress FPN and reduce the number of pixel output lines.

**Figure 3:**
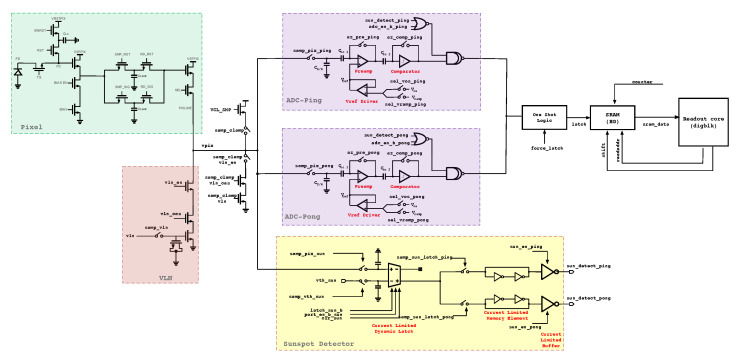
Complete analog signal chain (from pixel to readout core digital block).

To enable high frame rates, the row time must be minimized; this is achieved using a ping-pong single-slope ADC architecture, which pipelines sampling and ADC conversion. Figure 3 illustrates the analog readout signal chain of the image sensor. The authors refer readers to one of our previous works [11], which provides a comprehensive discussion of the challenges associated with high-speed ping-pong ADC architectures, with particular emphasis on minimizing electrical crosstalk between ADC pairs and mitigating differential nonlinearity (DNL) arising from high-speed counter distribution networks. In addition, one of our company white papers [12] presents detailed techniques for modeling and reducing low-light integral nonlinearity (INL) effects in ping-pong ADC architectures.

**Figure 4:**
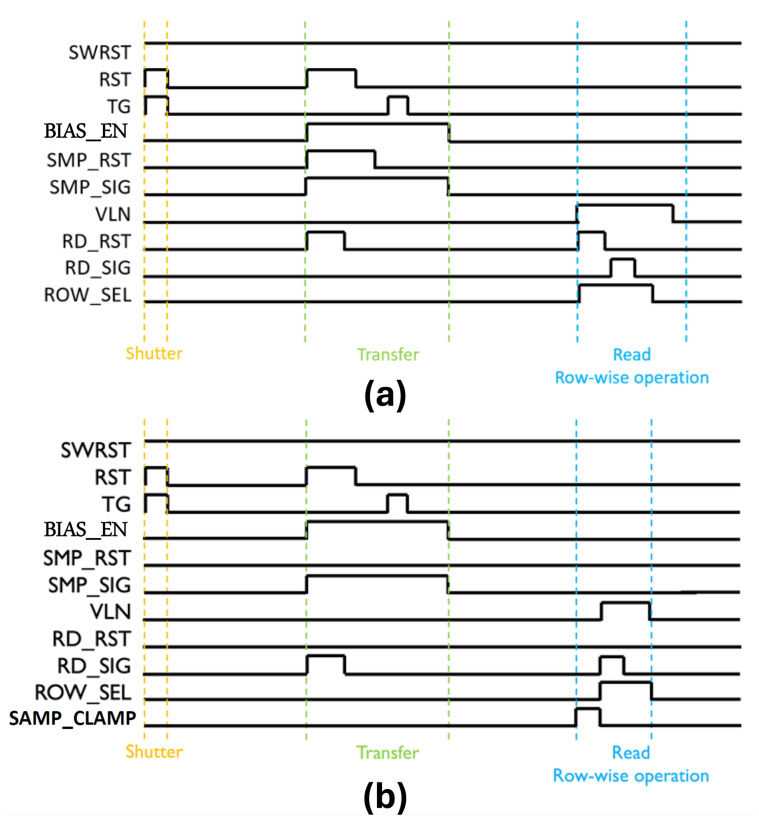
Simplified pixel timing for (**a**) CDS and (**b**) non-CDS operating modes.

In this implementation, the row time is primarily limited by the time required to sample both the reset and signal levels into the ADC during rolling readout. To trade off dark temporal noise for increased frame rate, a non-CDS readout mode is also supported, wherein a local ADC clamp can auto-zero the preamplifier and comparator—thereby reducing the reset sampling time. A high-level timing diagram illustrating pixel operation in CDS and non-CDS modes is shown in Figure 4. For the CDS timing, the read reset pulse during transfer phase is used to clear the residual charge on the parasitic capacitor at the gate of the output source follower. This helps to minimize any signal-dependent memory effect/lag on the next video frame. Please note that even though the pixel is inherently a dual gain pixel, we do not have a built-in dual gain mode implemented on-chip. The imager can either operate in high gain (SWRST always ON) or low gain (RST always ON) but not in interscenic or intrascenic dual gain mode.

**Figure 5:**
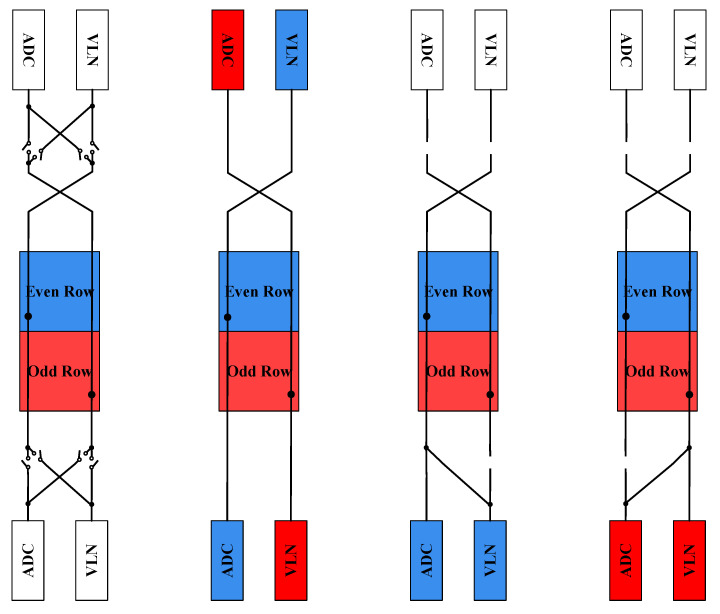
Illustration of the implemented VLN and ADC multiplexer network.

To support concurrent readout of six rows, each pixel column includes six pixlines. By default, even-numbered pixlines connect to the bottom readout chain, while odd-numbered pixlines connect to the top chain. To enable both dual-sided and single-sided readout modes—useful for port concentration—dynamically programmable switches are inserted along the even and odd pixlines. These switches allow each pixline to be routed to the ADC or VLN (or both), depending on the selected configuration as illustrated in Figure 5.

In dual-sided readout, the VLN associated with a given pixline resides on the opposite side from its ADC. In contrast, during single-sided readout, the programmable switches enable all pixlines to access both the ADC and VLN from the same side of the readout chain. One half of the readout circuitry can be completely powered down, resulting in significant power savings.

Flexible readout modes allow the sensor to output data through eight different port configurations. Larger resolutions will generate more pixel data, which can be read out faster using more output ports. Smaller resolutions will generate less pixel data and can be configured to use fewer output ports to save on power. This flexibility affords the sensor a variety of applications. The readout configuration is a function of two parameters: the resolution and desired number of output ports. The resolution determines how many superblocks are to be read out, and the number of output ports determines how the pixel data should be distributed.

**Figure 6:**
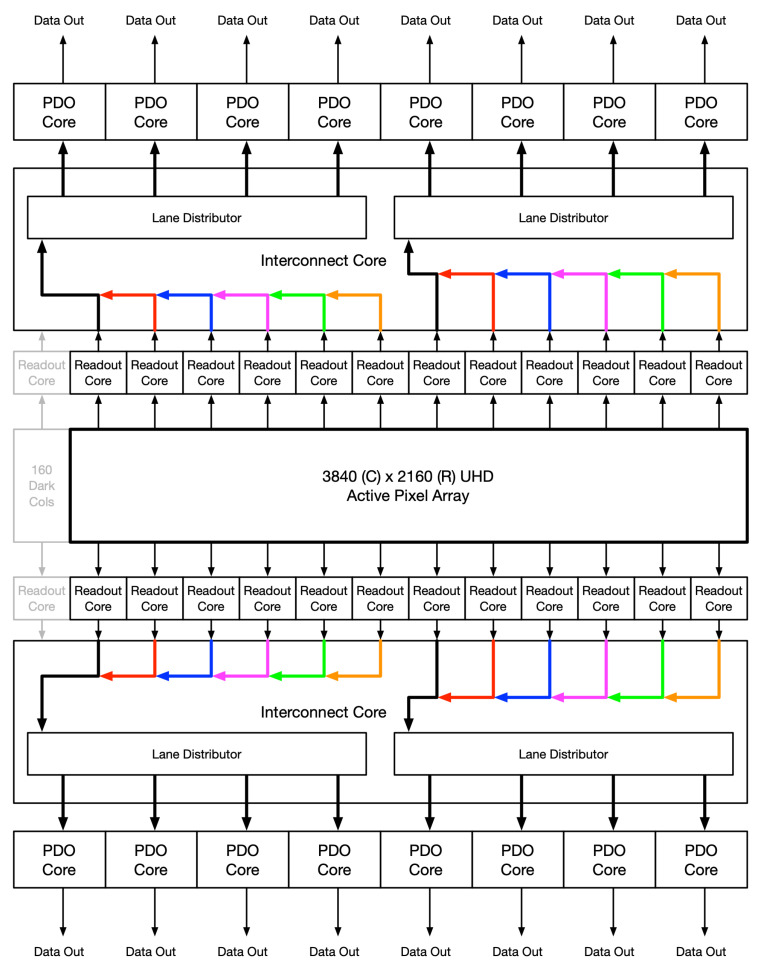
UHD resolution, 8 + 8 output ports.

All superblocks operate in parallel, each acquiring 320 columns by 3 rows worth of data. Each superblock writes their data into buffers immediately after acquiring the pixel data, while reading out from these buffers is done serially. After one buffer is emptied, the read operation points to the next buffer. A read controller determines which buffer to start reading from, when to transition to the next buffer, and the read rate. Figure 6 illustrates the flow of the pixel data through the digital data path for a UHD resolution.

**Figure 7:**
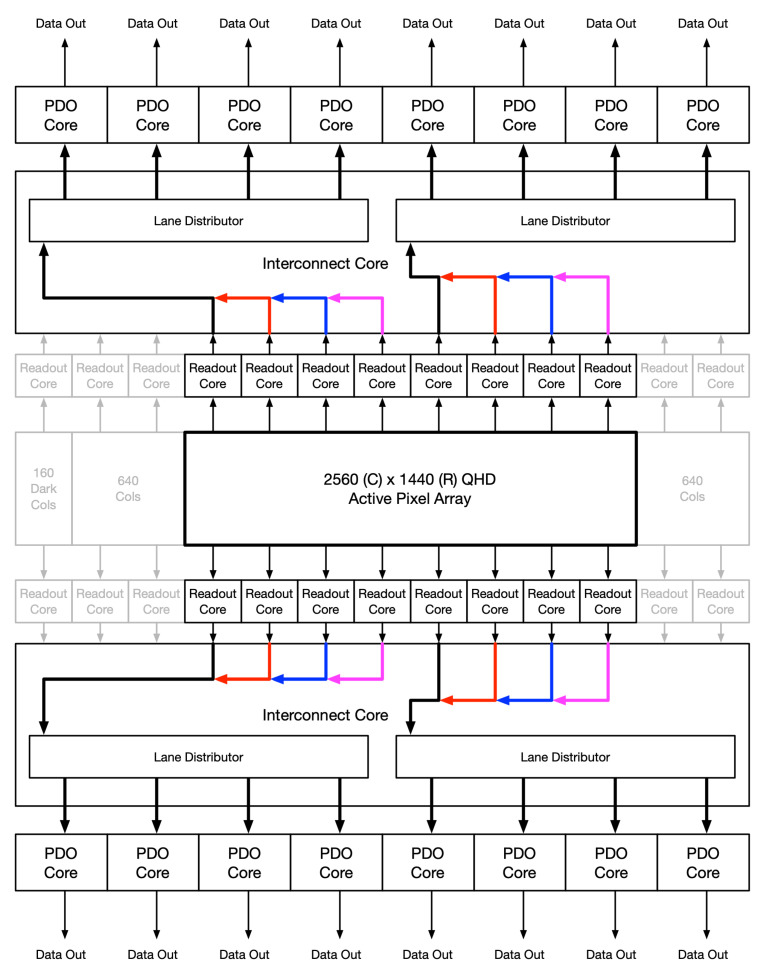
QHD resolution, 8 + 8 output ports.

**Figure 8:**
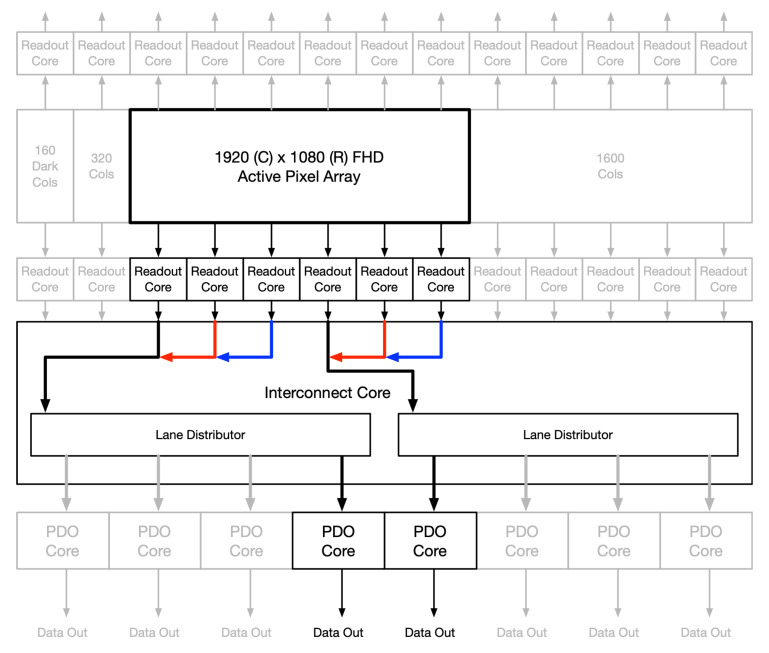
Off-centered FHD resolution, 2 output ports, single-sided readout.

The flexibility in the read controller allows windowed ROIs to still make use of all output data ports. Figure 7 shows the flow of the pixel data when windowed to QHD resolution. The data read from these buffers are streamed into two data channels. Each channel runs in parallel and always starts reading from the left buffer first. As each set of data comes in, it is evenly distributed to up to four output ports. If fewer ports are desired, then the channel controller will adjust the distribution. Figure 8 shows an off-centered FHD frame, outputting to only 2 ports in single-sided readout.

Once the data has been distributed to the output ports, framing information and meta data will be inserted into the data stream. This completed data stream will then be transformed using 64b/66b encoding to provide statistical bounds on DC balance, bit transition density, and allow for clock recovery. Flexibility in the readout configuration (resolution, windowing ROI, output bit depth, number of ports) is one the key features of the sensor’s data path.

One of the challenges of the digital block design comes from the implementation limitations, physical dimensions, and limited number of metal layers. The image sensor height is predominately made up of the pixel array and the readout circuitry through the top and bottom sides, leaving less than 1 mm each for the digital block height. This is further exacerbated by the width being over 21 mm, resulting in a very long and narrow shape. This can increase routing congestion since many signals that have to travel between the two ends will inevitably limit how local signals can move horizontally.

**Figure 9:**
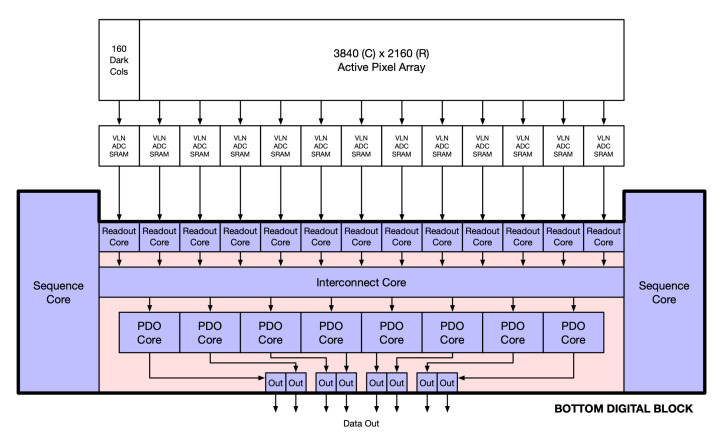
Bottom digital block floor plan.

In addition, various digital cores are implemented as macro blocks. This approach allows a single timing for the clean macro blocks to be arrayed out, while maintaining identical timing parameters. One downside is that the macros will block out any routing in the higher levels of hierarchy, keeping signals inside the macro strictly separate from signals outside of the macro. Many of the macros contain memory IP cores and so are limited in what kind of dimensions they can have. To address this limitation, certain digital control signals are routed through the macro blocks. This approach makes use of any available space in the macros but also eases the routing congestion outside. Figure 9 illustrates the connectivity between the different macros inside the digital block. The blue blocks represent macros, while the red areas depict the open space that can be used for routing and timing closure.

**Figure 10:**
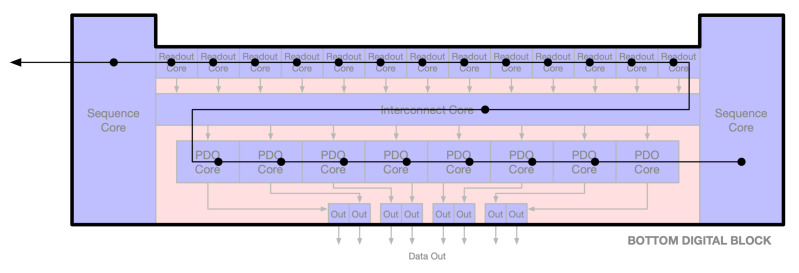
Bottom digital block floor plan-interface routing.

In Figure 9, the width of the readout cores and the interconnect core spanned nearly the full width of the digital block. The interconnect core was abutted to the sequence core meaning that there was no available path over or around the interconnect core. Anything passing between the readout core and the PDO cores had to first go through the interconnect core or the sequence cores. Certain interfaces, such as the digital test bus or communication bus, would have to snake through the digital block, as shown in Figure 10.

## 3. Row Logic

**Figure 11:**
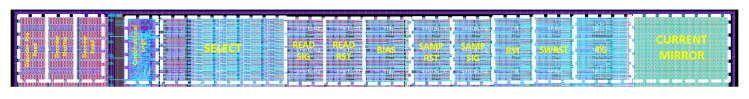
Row logic floor plan.

**Figure 12:**
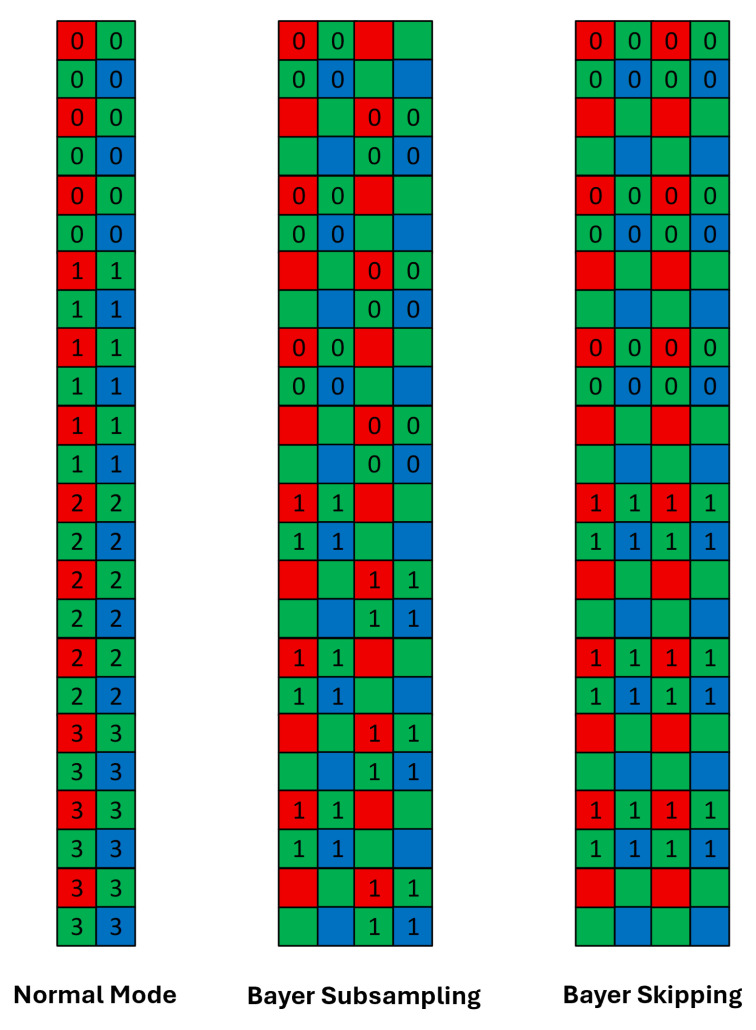
Pixel array column readout for the various spatial sub-sampling modes supported by the sensor. Note that 0, 1, 2, 3, etc., represent pixel sampling during first, second, third, and fourth row times, respectively.

The row-logic block (Figure 11) integrates the row decoders, spatial sub-sampling control logic, pixel-control signal generators, and current-mirror biasing network. Three independent decoders are implemented to generate the pre-read, shutter, and read pointers required for supporting both global-shutter and rolling-shutter readout modes. The associated combinational logic is synthesized to provide the necessary control stimuli for multiple spatial sub-sampling modes, including Bayer skipping and Bayer sub-sampling providing up to 2× frame rate boost (Figure 12).

A significant portion of the layout area is occupied by the nine dedicated pixel-control generators, which produce the key timing signals for each row: reset (RST), overflow (SWRST), transfer gate (TG), bias-enable (BIASSEL), sample-reset (SMP RST), sample-signal (SMP SIG), read-reset (RD RST), read-signal (RD SIG), and row-select (ROW SEL). Each generator incorporates a latch-based control circuit followed by a current-limited driver to ensure consistent edge shaping and robust signal delivery across the array.

The block also includes a current-mirror subsystem formed by repurposed pixel cells arranged as a bias-generation network. This structure provides a nominal 150-nA bias current to each pixel in the array, ensuring uniform operating conditions and minimizing pixel-to-pixel variation.

## 4. High-Speed Interface Design

Operating at UHD resolution at high frame rates, the image sensor generates a substantial volume of data, necessitating careful design, simulation, and optimization of the entire high-speed signal chain. To ensure seamless integration into the final camera system, several product-level constraints—such as a compact form factor, a reduced number of FPGAs in the data downlink, and low-power operation—shaped the high-speed clock distribution and SerDes architecture. The number of high-speed data ports was limited to 16 to align with system-level FPGA constraints, making it critical to maximize data throughput per port. Additionally, implementation on a 65nm process node with only four metal layers imposed further limitations on routing resources, driving the need for precise impedance control and signal integrity optimization across the high-speed paths.

**Figure 13:**
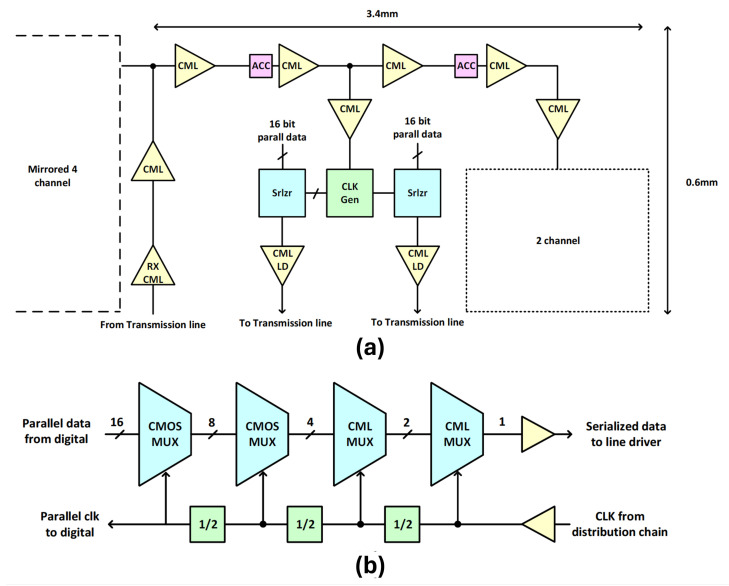
(**a**) High-speed signal path (from clock receiver to serialized data output), (**b**) high-speed 16-to-1 serializer architecture.

The high-speed signal chain comprises several key components, including the clock receiver, clock distribution network, serializer, and CML output driver (Figure 13a). A high-speed differential input clock operating at 3.72 GHz is supplied to both the top and bottom sides of the sensor readout. This clock is distributed through a high-speed CML-based clock distribution network to all eight CML output data ports—four located on the left and four on the right—ensuring synchronized data transmission across the entire high-speed interface.

To achieve UHD resolution at 12-bit depth and 1100 FPS, each of the 16 CML output ports (8 on the top and 8 on the bottom) operate at 7.44 Gbps. The clock receiver is a CML buffer comprising multiple amplification stages, designed to boost the small-swing differential input clock to a level sufficient for the clock distribution network. This receiver is placed at the center of a T-shaped clock tree, with each branch spanning 3.4 mm.

The number and placement of CML buffers along the clock distribution network are optimized through theoretical analysis—accounting for attenuation per unit trace length—and validated by post-layout simulations to ensure clock fidelity at each stage. A key challenge is common-mode imbalance in the differential clock, caused by random device mismatch and trace impedance variations. If uncorrected, this imbalance can degrade the differential signal, potentially leading to clock failure after several stages.

To mitigate this, AC coupling is introduced periodically in the clock path. While effective in restoring common-mode balance and filtering low-frequency noise from earlier stages, AC coupling introduces additional parasitic capacitance due to the coupling capacitor (realized using MOS devices) and the input capacitance of the CML stage. This imposes a practical limit on the number of AC coupling stages. The high-pass filter formed by the AC capacitor and bias resistor is designed with a cutoff frequency at one-fourth of the operating clock frequency. However, larger capacitors add parasitic loading, and higher-value resistors increase thermal noise, necessitating careful sizing trade-offs to balance signal integrity and noise performance.

To further minimize substrate-related uncertainties, additional metal shielding is routed beneath the high-speed signal traces to provide controlled coupling and reduce undesired coupling to the substrate. While layout extraction tools often model the substrate as a zero-impedance ground, in practice, it exhibits a distributed RC behavior that depends heavily on layout geometry and process parameters. To mitigate these effects, the thick top metal layer—available in the 4-metal process—is utilized for routing critical signals, thereby reducing resistance and parasitic capacitance. This approach helps maintain stable duty cycles, which is essential for robust DDR operation of the serializer.

As shown in Figure 13a,b, the clock generation block produces a divided clock for the 16:1 serializer, shared between two serializer units placed symmetrically on the left and right sides. High-speed clock domains utilize CML-type dividers, whereas CMOS TSPC-based dividers are employed for low-speed clock generation. In stages 1 and 2, the divided clock signals are converted to CMOS levels. The stage 1 clock is also used as a parallel clock routed to the digital block. This ensures synchronization between the digital parallel data and the serializer input.

The serializer is structured as a multi-stage multiplexer (MUX) tree optimized for both performance and area efficiency:

- **CMOS Stages:** The first stage consists of eight units of 2:1 CMOS MUXes implemented using TSPC logic for low-power, compact operation at low frequencies followed by the second stage with four units of 2:1 CMOS MUXes to further serialize data.
- **CML Stages:** The third stage has two units of 2:1 CML MUXes followed by the fourth stage consisting of just one unit of 2:1 CML MUX with a one-clock delayed data path generated to support post-emphasis functionality, which improves signal integrity at high data rates.

The serializer output connects to a CML output driver with a 50 Ω resistive load for impedance matching with the transmission line. An integrated post-emphasis circuit with programmable coefficients compensates for channel loss, enhancing high-frequency signal quality. To ensure robustness, reverse-biased diodes are placed at each differential output for ESD protection. Additionally, RLC elements are inserted in the supply and ground paths during post-layout simulations to model bonding wire effects. The transmission channel is characterized using S-parameter data to accurately capture high-speed behavior and guarantee signal integrity.

## 5. Results and Summary

Table 1 and Table 2 summarize the scaling of maximum achievable frame rates and power consumption for various standard video resolutions for different bit depths and various port configurations for the CDS and non-CDS modes, respectively. The sensor’s readout architecture is designed to support higher frame rates when operating at reduced resolution formats. Across the supported video formats and output-port configurations, the maximum achievable frame rate generally increases as the ADC bit-depth is reduced from 12-bit to 10-bit and further to 8-bit. For the UHD format, the frame rate is consistently constrained by the total available output bandwidth, regardless of bit-depth. In contrast, for lower-resolution formats, the limiting factor depends on both ADC bit-depth and the number of active output ports. At 12-bit resolution, the ADC conversion time—set by the counter speed—typically governs the maximum frame rate. At 10-bit and 8-bit resolutions, the reset and signal sampling phases dominate the row time and therefore become the primary bottleneck instead of ADC conversion.Additionally, when operating with fewer output ports, the reduced output bandwidth can become increasingly limiting as the video resolution increases. In such cases, bandwidth constraints may dominate over sampling and ADC-conversion limits, particularly for higher-resolution video modes.

**Figure 14:**
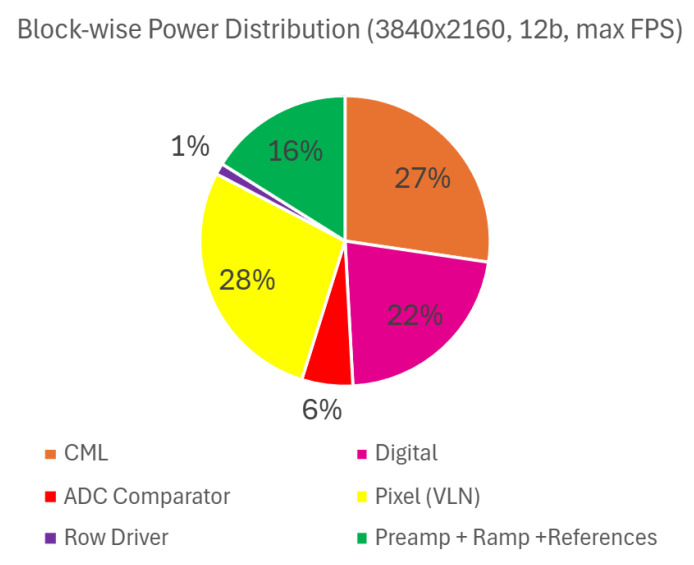
Block-wise power breakdown at UHD resolution, 12b, and max frame rate.

**Figure 15:**
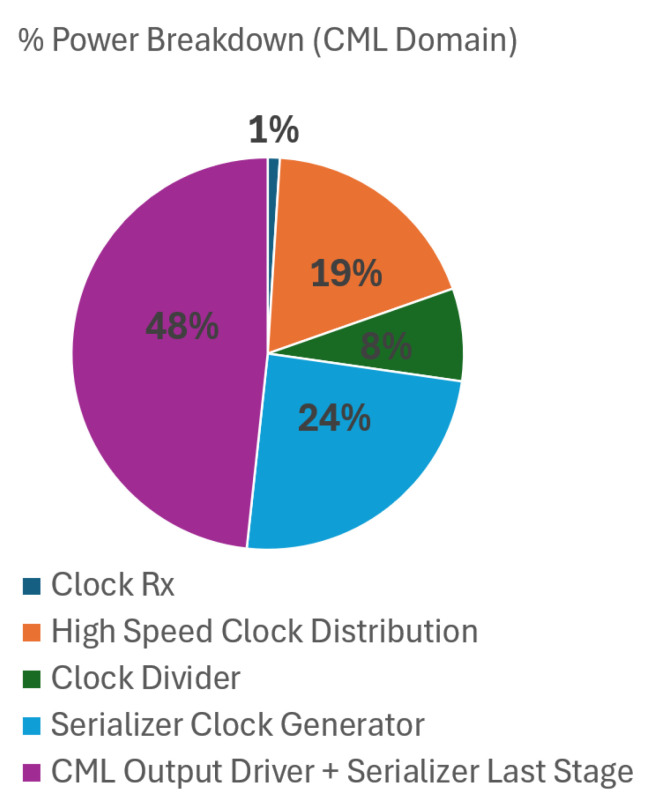
Block-wise CML power breakdown at UHD resolution, 12b, and max frame rate.

A noticeable reduction in total power consumption is observed when fewer output data ports are used. In dual-sided readout mode, the savings primarily stem from disabling unused serializers and output drivers. In single-sided readout mode, additional power reduction is achieved by powering down the entire readout circuitry on the inactive side, including VLN, ADCs, SRAMs, digital logic, clock distribution, serializers, and output drivers. This selective shutdown of components contributes significantly to overall power efficiency. Figure 14 illustrates the relative contribution of major sub-blocks to the overall power consumption, presented as a percentage breakdown. Figure 15 shows the major contributors to the CML domain power consumption. To optimize power consumption for application-specific frame rate targets, the number of active output data ports can also be reduced without sacrificing performance.

**Figure 16:**
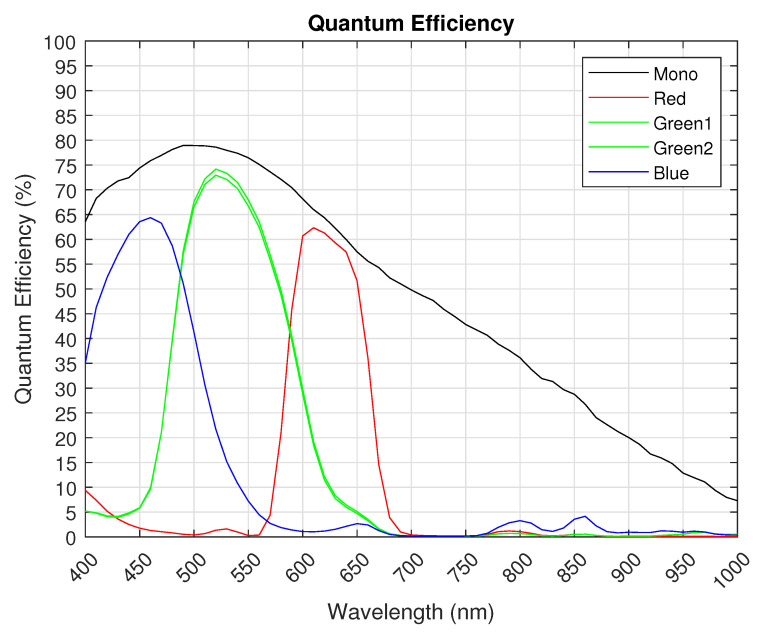
Quantum efficiency vs. wavelength (in nm).

**Figure 17:**
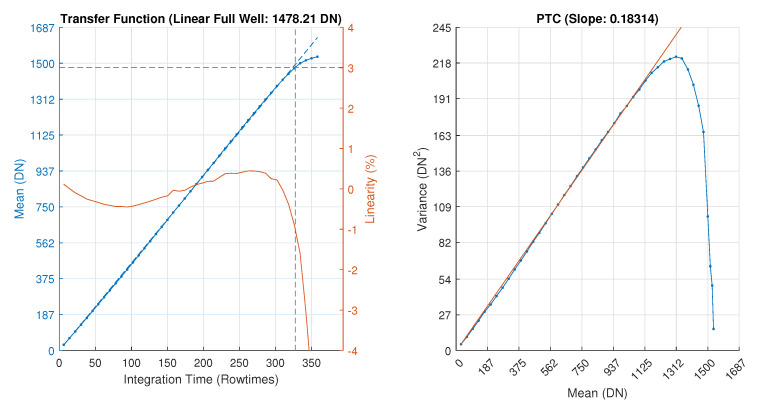
Transfer function linearity and photon transfer curve (PTC).

**Figure 18:**
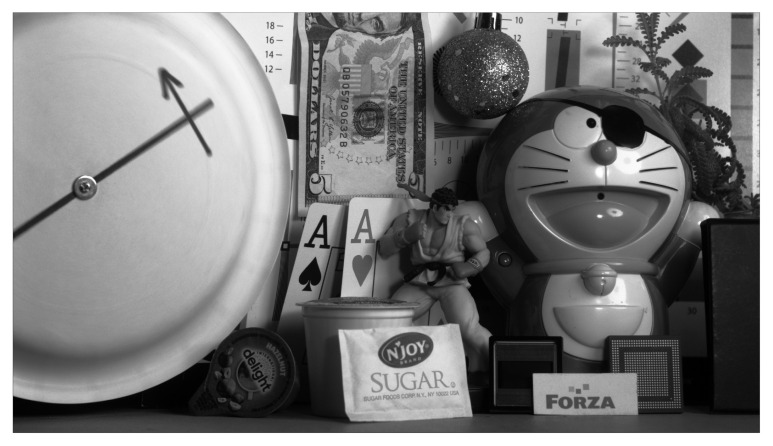
Monochromatic capture at 12-bit resolution at 1100 FPS.

Detailed specifications of the CIS sensor are presented in Table 3 along with QE curves for mono and RGGB CFA in Figure 16. The Photon Transfer Curve (PTC) and transfer function linearity are shown in Figure 17. A full-resolution 12-bit monochrome image captured at 1100 FPS is shown in Figure 18.

Table 4 presents a performance comparison of the proposed imager (ForzaFAST581) with other publicly available high-speed global shutter CIS devices of comparable UHD/4K resolution. While every effort has been made to align the specifications across sources, it should be noted that many manufacturers do not disclose complete parameter sets, and that some of the compared sensors may have been primarily designed for applications other than machine vision. The proposed imager demonstrates state-of-the-art performance in terms of noise and parasitic light sensitivity (PLS). In 12-bit mode, it achieves more than twice the frame rate of [13] while utilizing nine times fewer output ports, thereby enabling more compact camera integration, and provides an improvement of more than 14 dB in PLS. Relative to [14], the imager delivers over a twofold increase in frame rate (at 10-bit operation) together with approximately fifteen times lower noise. The proposed imager attains a slightly lower maximum frame rate than [15]; however, since no noise or linearity data are publicly available for that sensor, it remains unclear whether its higher frame rate is achieved at the expense of noise performance or overall image quality.

## Figures and Tables

**Table 1:** Frame rate and power consumption scaling vs. bit-depth across various port configurations (CDS mode).

Resolution	Bit-Depth	8 Top + 8 Bot	6 Top + 6 Bot	4 Top + 4 Bot	2 Top + 2 Bot	8 [One-Side]	6 [One-Side]	4 [One-Side]	2 [One-Side]
3840 × 2160 (UHD)	*12-bit*	1141 (5.51W) Data Rate Limited	857 (5.24W) Data Rate Limited	573 (3.98W) Data Rate Limited	287 (3.24W) Data Rate Limited	573 (2.70W) Data Rate Limited	430 (2.45W) Data Rate Limited	287 (1.92W) Data Rate Limited	144 (1.66W) Data Rate Limited
	*10-bit*	1359 (5.41W) Data Rate Limited	1026 (5.23W) Data Rate Limited	688 (3.98W) Data Rate Limited	344 (3.24W) Data Rate Limited	682 (2.30W) Data Rate Limited	515 (2.57W) Data Rate Limited	345 (2.00W) Data Rate Limited	172 (1.70W) Data Rate Limited
	*8-bit*	1694 (5.39W) Data Rate Limited	1281 (4.96W) Data Rate Limited	859 (3.77W) Data Rate Limited	430 (3.13W) Data Rate Limited	850 (3.00W) Data Rate Limited	642 (2.56W) Data Rate Limited	431 (2.00W) Data Rate Limited	215 (1.70W) Data Rate Limited
2560 × 1440 (QHD)	*12-bit*	2199 (5.84W) ADC Conversion Limited	1909 (5.18W) Data Rate Limited	1280 (4.22W) Data Rate Limited	644 (3.25W) Data Rate Limited	1106 (3.10W) ADC Conversion Limited	960 (2.48W) Data Rate Limited	644 (1.98W) Data Rate Limited	323 (1.63W) Data Rate Limited
	*10-bit*	3024 (5.83W) Data Rate Limited	2294 (5.16W) Data Rate Limited	1533 (4.21W) Data Rate Limited	773 (3.25W) Data Rate Limited	1524 (3.32W) Data Rate Limited	1154 (2.62W) Data Rate Limited	771 (2.10W) Data Rate Limited	388 (1.68W) Data Rate Limited
	*8-bit*	3543 (5.48W) Sampling Time Limited	2847 (4.86W) Data Rate Limited	1917 (3.95W) Data Rate Limited	964 (3.10W) Data Rate Limited	1782 (3.32W) Sampling Time Limited	1432 (2.62W) Data Rate Limited	962 (2.09W) Data Rate Limited	484 (1.68W) Data Rate Limited
1920 × 1080 (Full HD)	*12-bit*	2920 (5.65W) ADC Conversion Limited	2920 (5.54W) ADC Conversion Limited	2264 (4.22W) Data Rate Limited	1141 (3.39W) Data Rate Limited	1472 (3.02W) ADC Conversion Limited	1472 (2.64W) ADC Conversion Limited	1141 (2.09W) Data Rate Limited	573 (1.66W) Data Rate Limited
	*10-bit*	4254 (5.64W) Sampling Time Limited	4011 (5.30W) Data Rate Limited	2697 (4.10W) Data Rate Limited	1369 (3.39W) Data Rate Limited	2150 (3.25W) Sampling Time Limited	2027 (2.82W) Data Rate Limited	1359 (2.12W) Data Rate Limited	688 (1.73W) Data Rate Limited
	*8-bit*	4705 (5.55W) Sampling Time Limited	4705 (5.20W) Sampling Time Limited	3361 (4.09W) Data Rate Limited	1710 (3.20W) Data Rate Limited	2372 (3.25W) Sampling Time Limited	2372 (2.84W) Sampling Time Limited	1694 (2.12W) Data Rate Limited	859 (1.73W) Data Rate Limited
1280 × 720 (HD)	*12-bit*	4345 (5.46W) ADC Conversion Limited	4345 (5.36W) ADC Conversion Limited	4345 (4.68W) ADC Conversion Limited	2530 (3.77W) Data Rate Limited	2199 (2.94W) ADC Conversion Limited	2199 (2.56W) ADC Conversion Limited	2199 (2.24W) ADC Conversion Limited	1280 (1.78W) Data Rate Limited
	*10-bit*	6313 (5.45W) Sampling Time Limited	6313 (5.11W) Sampling Time Limited	5952 (4.66W) Data Rate Limited	3029 (3.77W) Data Rate Limited	3208 (3.16W) Sampling Time Limited	3208 (2.75W) Sampling Time Limited	3024 (2.42W) Data Rate Limited	1533 (1.89W) Data Rate Limited
	*8-bit*	7000 (5.36W) Sampling Time Limited	7000 (5.01W) Sampling Time Limited	7000 (4.32W) Sampling Time Limited	3803 (3.51W) Data Rate Limited	3543 (3.16W) Sampling Time Limited	3543 (2.75W) Sampling Time Limited	3543 (2.43W) Sampling Time Limited	1917 (1.89W) Data Rate Limited
640 × 480 (VGA)	*12-bit*	6439 (5.27W) ADC Conversion Limited	6439 (5.16W) ADC Conversion Limited	6439 (4.47W) ADC Conversion Limited	6439 (3.61W) ADC Conversion Limited	3279 (2.86W) ADC Conversion Limited	3279 (2.48W) ADC Conversion Limited	3279 (2.16W) ADC Conversion Limited	3279 (1.70W) ADC Conversion Limited
	*10-bit*	9319 (5.26W) Sampling Time Limited	9319 (4.92W) Sampling Time Limited	9319 (4.47W) Sampling Time Limited	8786 (3.60W) Sampling Time Limited	4773 (3.08W) Data Rate Limited	4773 (2.67W) Sampling Time Limited	4773 (2.35W) Sampling Time Limited	4500 (1.81W) Data Rate Limited
	*8-bit*	10,374 (5.17W) Sampling Time Limited	10,374 (4.82W) Sampling Time Limited	10,374 (4.12W) Sampling Time Limited	10,374 (3.34W) Sampling Time Limited	5282 (3.08W) Sampling Time Limited	5282 (2.67W) Sampling Time Limited	5282 (2.35W) Sampling Time Limited	5282 (1.81W) Sampling Time Limited

**Table 2:** Frame rate scaling vs. bit-depth across various port configurations (non-CDS mode).

Resolution	Bit-Depth	8 Top + 8 Bot	6 Top + 6 Bot	4 Top + 4 Bot	2 Top + 2 Bot	8 [One-Side]	6 [One-Side]	4 [One-Side]	2 [One-Side]
3840 × 2160 (UHD)	*12-bit*	1144	859	573	287	574	431	287	144
	*10-bit*	1363	1029	688	344	683	516	345	172
	*8-bit*	1699	1281	859	430	851	642	431	215
2560 × 1440 (QHD)	*12-bit*	2208	1917	1286	644	1108	962	645	323
	*10-bit*	3037	2303	1539	773	1528	1156	773	388
	*8-bit*	3796	2859	1917	964	1910	1435	962	484
1920 × 1080 (Full HD)	*12-bit*	2936	2936	2277	1141	1476	1476	1144	573
	*10-bit*	5360	4033	2712	1369	2702	2033	1363	688
	*8-bit*	6515	5041	3379	1710	3284	2541	1699	859
1280 × 720 (HD)	*12-bit*	4381	4381	4381	2551	2208	2208	2208	1286
	*10-bit*	9693	8874	6000	3054	4906	4491	3037	1539
	*8-bit*	9693	9693	7500	3803	4906	4906	3796	1917
640 × 480 (VGA)	*12-bit*	6518	6518	6518	6518	3299	3299	3299	3299
	*10-bit*	14,365	14,365	14,365	8892	7314	7314	7314	4528
	*8-bit*	14,365	14,365	14,365	11,115	7314	7314	7314	5660

**Table 3:** Specification table for ForzaFAST581.

Parameter	Specification
Technology	1P4M, 65 nm BSI
Resolution	3840 × 2160 (Mono and Color)
Pixel Pitch	5 µm
Shutter Type	Voltage Domain Global Shutter
Linear Full Well	8100 e^−^ (HG), 41,300 e^−^ (LG) (LG full well Photodiode limited)
Conversion Gain	170 µV/e^−^ (HG), 19.3 µV/e^−^ (LG)
Total Dark Temporal Noise (HG)	CDS: 3.04 e^−^ (GS), 2.15 e^−^ (RS) non-CDS: 12.7 e^−^ (GS)
Row Noise Correction	On-Chip
Row Noise	0.46 e^−^ without correction 0.2 e^−^ with correction
Max Frame Rate	215,277 FPS with CDS (640 × 6)
Minimum Exposure Time	1.77 µs
ADC Resolution	8 b/10 b/12 b
ADC Counter Speed	2.48 GHz gray counter
PRNU	2.2% (HG), 2.1% (LG)
DSNU	14.6 e^−^ (HG), 128 e^−^ (LG)
Dynamic Range	68 dB (HG)
Max SNR	39 dB (HG)
Quantum Efficiency	65%@430 nm (blue) 73%@530 nm (green) 61%@620 nm (red) 80%@500 nm (mono)
PLS	<−100 dB measured @658 nm
Power Consumption	5.5 W
Data Ports	16 × outputs @ 7.44 Gbps
Die Size	23.55 mm (H) × 25.6 mm (V)
Trigger Control	Internal and External
Serial Interface	SPI

**Table 4:** Performance comparison of ForzaFAST581 with other publicly available high-speed global shutter CIS chips. HG = high gain, LG = low gain, PLS = parasitic light sensitivity, N/A = data not available.

Parameter	This Work (ForzaFAST581)	GSPRINT 4510 [13]	LUX8M [16]	LUX9512 [17]	IMX926 AQJ [18]	ams CMV12000 [19]	Chronos 4K12 [20]	Lince 11M [14]	Photron FASTCAM MINI R5-4K [15]
Pixel Pitch (μm)	5	4.5	5	6.5	2.74	5.5	4.5	6	6.5
Shutter Type	Global (Voltage) and Rolling	Global (Charge)	Global	Global	Global	Global	Global	Global	Global
Resolution	3840 × 2160	4608 × 2176	3904 × 2192	4096 × 2304	4128 × 3072	4096 × 3072	4096 × 2160	4480 × 2496	4096 × 2304
Max Frame Rate (FPS)	1141/1359/ 1694@ 12/10/8b	480/1000/ 1920@ 12/10/8b	264@12b	1333@full res	318.6/588.8/ 660.8@ 12/10/8b	300@10b	481/1006/ 1491@ 12/10/8b	609	1250
Output Data	16×@7.44 Gbps	144 ch Sub-LVDS@1.2 Gbps	32 LVDS@0.9 Gbps	128 LVDS	8×2 SLVS-EC@12.474 Gbps	64 LVDS@ 600 Mbps	N/A	N/A	N/A
Full Well Capacity (e^−^)	8100 (HG); 41,300 (LG)	>30k (LG)	11.5k (HG)	12k (HG)	N/A	13.5k	N/A	45k	N/A
Dark Noise (e^−^)	3.04 (HG)	<4	10	8	N/A	13	N/A	45	N/A
Dynamic Range (dB)	68	68	N/A	N/A	>66	60	N/A	60	N/A
Power (W)	5.5	2.5	3.83@264 FPS	5.7	N/A	4.2 (max)	N/A	3.6	N/A
PLS (dB)	<−100	<−86	N/A	N/A	N/A	N/A	−94	N/A	N/A
Bit Resolution	12/10/8	12/10/8	12	12/10/8	12/10/8	12/10/8	12/10/8	10	12
Min Integration Time (μs)	1.77	N/A	N/A	N/A	1	15.4	1	N/A	2

## Data Availability

The original contributions presented in this study are included in the article. Further inquiries can be directed to the corresponding author.
